# Draft Genome Sequences of the Clinical Isolates Kp 23 and KPM 20

**DOI:** 10.1128/MRA.00119-21

**Published:** 2021-03-25

**Authors:** Alyssa K. W. Maclean, Nancy D. Hanson

**Affiliations:** a Department of Medical Microbiology and Immunology, Creighton University School of Medicine, Omaha, Nebraska, USA; b Center for Research in Anti-Infectives and Biotechnology, Creighton University School of Medicine, Omaha, Nebraska, USA; University of Maryland School of Medicine

## Abstract

Klebsiella pneumoniae strains are capable of becoming resistant through multiple mechanisms. Here, we announce draft sequences for Kp 23, a clinical isolate with no plasmid-encoded β-lactamases, and KPM 20, a clinical isolate with no plasmid-encoded β-lactamases and no detectable OmpK35, OmpK36, or PhoE in the outer membrane.

## ANNOUNCEMENT

Klebsiella pneumoniae confers multidrug resistance through the loss of the outer-membrane porins OmpK35, OmpK36, and PhoE (1–3). However, many of these isolates retain intact structural genes that encode full-length porin proteins (4, 5). While an important factor in multidrug resistance, the exact contribution of porin loss when combined with other resistance mechanisms is unknown. This genome announcement describes the whole-genome sequences of Kp 23, a reference strain in our laboratory collected in 1973, and KPM 20, a cephalosporin-susceptible strain lacking OmpK35, OmpK36, and PhoE in the outer membrane.

Cultures were grown in Mueller-Hinton broth (MHB) at 37°C with shaking. DNA was extracted with the Qiagen MagAttract kit following manufacturer instructions. DNA was quantified using Qubit with the high-sensitivity (HS) DNA assay. Libraries were prepared using the Illumina Nextera Flex DNA kit with 250 ng DNA, optimized for 300-bp paired-end reads (6). Sequencing was performed on the Illumina MiSeq platform. Low-quality reads were filtered and Nextera adapter sequences removed using TrimmomaticPE version 0.36 (7).

The genomes were assembled as previously described by Harrison and Hanson (8, 9). Default parameters were used for all software unless otherwise specified. Initial contig libraries were created using SPAdes version 3.14.1 with the parameters “-k 21, 33, 55, 77, 88, 127,” “-careful,” and “-cov-cutoff 5” (10) and SGA version 0.10.15 with default parameters and “-pe-mode” set to 1 (11). These assemblies were merged using SPAdes and ordered with Ragout version 2.0 (12) using reference genomes (Kp 23, GenBank accession numbers CP021165.1, CP020071.1, CP018671.1, and CP025639.1; KPM 20, CP061700.1, CP056216.1, and CP024542.1). The contigs were joined using AlignGraph version 1.0 with the “distanceLow” and “distanceHigh” parameters set to 300 and 1,602, respectively (13). Using this assembly as a scaffold and the initial trimmed sequence files, a final reference-guided genome assembly was generated using SPAdes. Contigs less than 500 bases were filtered. The genomes were annotated using the NCBI Prokaryotic Genome Annotation Pipeline version 5.0 and further assessed using PlasmidFinder version 4.1 (14) and ResFinder version 2.1 (15).

The assembly statistics for Kp 23 and KPM 20 are listed in Table 1. Upon annotation, the Kp 23 genome was found to contain 5,518 genes encoding 5,293 protein-coding sequences, 120 pseudogenes, 79 tRNAs, 12 rRNAs, and 14 noncoding RNAs (ncRNAs). The plasmid sequences were consistent with IncFIB(K), IncFII(K), and IncFII(pKP91). The resistance genes were *bla*_SHV-40_, *fosA*, *oqxA*, and *oqxB*. Annotation of the KPM 20 assembly showed 5,540 genes, with 5,257 protein-coding sequences, 183 pseudogenes, 78 tRNAs, 11 rRNAs, and 11 ncRNAs. The plasmid sequences Col(MG828), Col(pHAD28), and IncFIB(K)(pCAV1099-114) were identified. The resistance genes *bla*_SHV-56_, *fosA*, *oqxA*, and *oqxB* were detected.

**TABLE 1:** Assembly statistics for Kp 23 and KPM 20

Isolate	No. of reads	Genome size (bp)	No. of contigs	G+C content (%)	*N*_75_ (bp)	*N*_50_ (bp)	*L*_75_	*L*_50_	Accession no.
Kp 23	4,459,218	5,596,095	58	57.2	168,132	361,162	11	5	JAEVHE000000000
KPM 20	4,451,906	5,562,045	119	57.08	64,450	134,417	25	11	JAEVHD000000000

K. pneumoniae resistance can emerge through the loss of outer membrane porins. Here, we provide two genome sequences with similar acquired resistance genes. Kp 23 serves as a useful reference for genome assemblies and as a comparator strain for further research. KPM 20 provides the genome sequence of a porin-deficient strain to examine the role of porins in antimicrobial resistance.

### Data availability.

The whole-genome shotgun assemblies have been deposited at DDBJ/ENA/GenBank under the BioSample accession numbers JAEVHE000000000 and JAEVHD000000000 for Kp 23 and KPM 20, respectively. The versions described in this paper are JAEVHE010000000 and JAEVHD010000000, respectively. The raw data can be accessed under the SRA accession numbers SRX9980334 and SRX9980335 for Kp 23 and KPM 20, respectively.
